# A Surgical Presentation of Churg-Strauss Syndrome

**DOI:** 10.7759/cureus.24342

**Published:** 2022-04-21

**Authors:** Nikhil Vasandani, Martha Isaac, Amrit Bajwa, Margaret Sheehan, Emmeline Nugent

**Affiliations:** 1 Department of Surgery, University Hospital Galway, Galway, IRL; 2 Department of Pathology, University Hospital Galway, Galway, IRL

**Keywords:** churg-strauss syndrome, high-dose methylprednisolone, mepolizumab, impending pericardial effusion, acute rheumatology, intermittent claudication, pulmonary sarcoidosis, internal medicine and rheumatology, acute generalised peritonitis, surgical acute abdomen

## Abstract

Eosinophilic granulomatosis with polyangiitis (EGPA) or Churg-Strauss syndrome (CSS) is a rare, autoimmune vasculitis usually affecting small and medium-sized blood vessels in its later phases. It is a diffuse, systemic, multisystem disease that is reported to present with gastrointestinal manifestations but very rarely as an acute abdomen secondary to eosinophilic peritonitis. A 28-year-old relatively healthy male with a pre-existing diagnosis of inactive pulmonary sarcoidosis presented to the emergency department with an acute abdomen. After an exploratory laparotomy, multi-specialty involvement, and extensive investigations to exclude other differentials, a diagnosis of EGPA was made. The patient was treated with systemic glucocorticoids initially, followed by a tapering course of steroids and anti-interleukin 5 monoclonal antibodies as maintenance upon remission. EGPA can manifest in a myriad of ways including an acute abdomen, and medical treatment is useful in managing this presentation. Surgeons should be aware of the atypical causes of acute abdomen and should routinely broaden their differential diagnosis to include medical pathologies.

## Introduction

Eosinophilic granulomatosis with polyangiitis (EGPA) or Churg-Strauss syndrome (CSS) is a diffuse, systemic, multisystem disease primarily affecting but not limited to the lungs, usually in individuals with atopic history. The lungs are the most commonly involved organ system followed by the skin. EGPA can affect the cardiovascular, gastrointestinal, renal, and central nervous systems, with higher rates of mortality and morbidity reported in those with vasculitis and multisystem involvement [1].

Historically, EGPA was first termed “allergic granulomatosis and angiitis” in 1951 by pathologists Jacob Churg and Lotte Strauss to describe a pattern of illness seen in 13 autopsied patients, most of whom were initially diagnosed with polyangiitis nodosa. This subset of patients presented with severe asthma, hypereosinophilia, and evidence of granulomatous necrotizing vasculitis affecting multiple organs. In addition to the clinical findings, pathological examination revealed extravascular granulomas, tissue eosinophilia, and necrotizing vasculitis. The presence of all three pathological findings became the diagnostic criteria for this distinctive form of vasculitis later titled “Churg-Strauss syndrome” [2].

Epidemiologically speaking, the prevalence of EGPA in Europe ranges anywhere from 10.7 to 14 per million. Approximately 10% of patients with a major form of vasculitis are recognized to have EGPA [3]. A cross-sectional study performed by the UK and Ireland vasculitis registry revealed the mean age at diagnosis of EGPA to be 55.8 years with 90% being Caucasians and a greater female to male preponderance. EGPA is also rare in children and adolescents; when it does occur in this age group, it appears to follow a more aggressive course with prominent pulmonary and cardiovascular manifestations. Amongst the Irish population, the organ system most commonly involved is the respiratory system, closely followed by the ENT, mucocutaneous, and peripheral nervous system, respectively [4].

Diagnosing CSS is very robust. In 1990, the American College of Rheumatology (ACR) established diagnostic criteria for CSS, which boasted a 99.7% specificity and 85% sensitivity. These criteria require patients to meet four out of six following features. These include asthma, migratory infiltrates in the lung, a paranasal sinus abnormality, mono or polyneuropathy, peripheral blood eosinophilia, and eosinophilic tissue infiltrates in biopsies [2]. The updated 2022 criteria were recently published by the ACR in association with the European Alliance of Associations for Rheumatology (EULAR). These criteria are points-based and assign certain features that support or negate an EGPA diagnosis with positive and negative points, respectively. These include a maximum eosinophil count of ≥ 1 × 10^9/L to be given five points, evidence of obstructive airway disease to be assigned three points, evidence of nasal polyps with three points, and being cytoplasmic antineutrophil cytoplasmic antibody (ANCA) or anti-proteinase 3 (anti-PR3) ANCA positive with negative three points. Evidence of extravascular eosinophilic predominant inflammation is given two points, diagnosis of mononeuritis multiplex/motor neuropathy not due to radiculopathy is one point, and evidence of hematuria is assigned negative one point. Diagnosis of EGPA was supported by a cumulative score of ≥ six points. When these criteria were tested in the validation dataset, the sensitivity was 85% (95% CI: 77-91%) and the specificity was 99% (95% CI: 98-100%) [5].

## Case presentation

**Figure 1:**
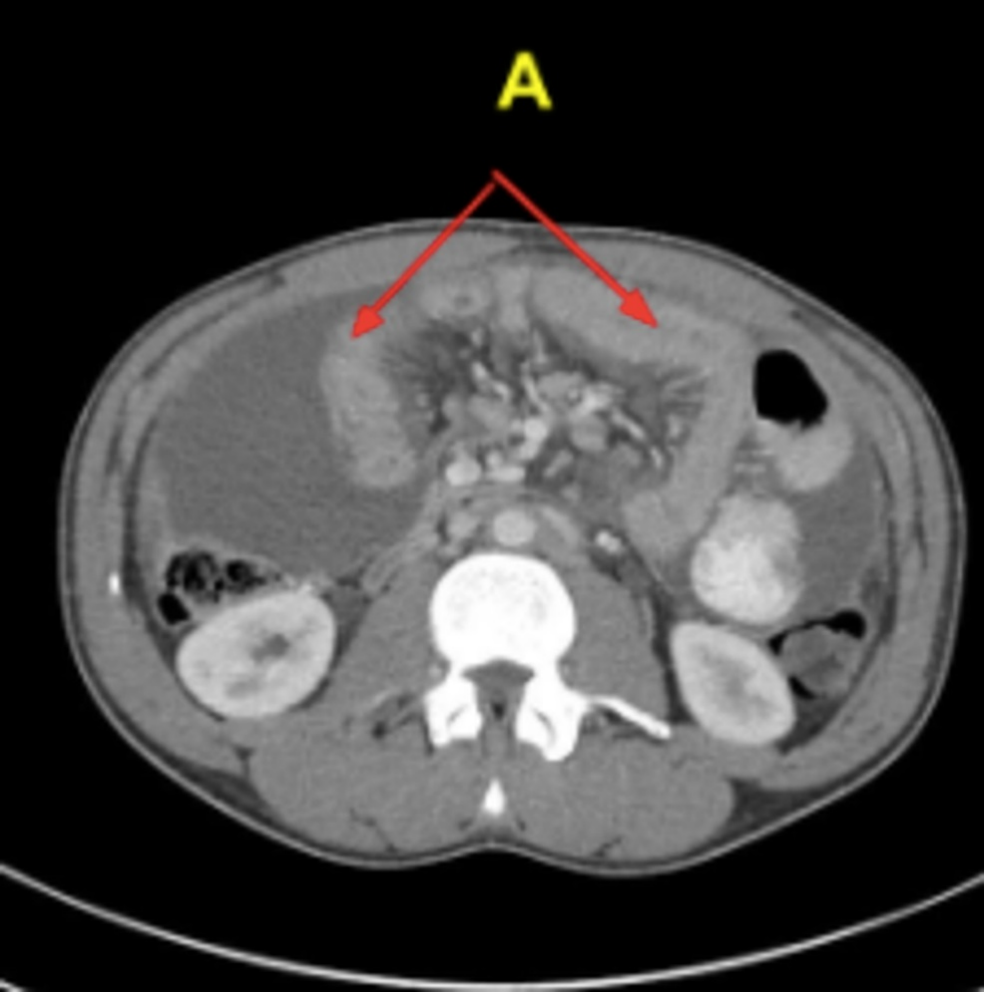
Axial CT image showing mural thickening and diffuse edema of the small bowel, most notably the jejunum (marked by red arrows, labeled A).

The patient was a 28-year-old male who presented to the emergency department with sharp, gradual onset, worsening, diffuse abdominal pain for the past week, which was being managed conservatively as reflux by the general practitioner with failure. He described the pain to be 9/10 in terms of severity, which radiated to the back and was exacerbated with movement. This presentation was accompanied by a period of anorexia and nausea for approximately five days. The patient denied any episodes of vomiting, diarrhea, pyrexia, constipation, rectal bleeds, or melena. He reported normal bowel movements and the ability to pass flatus. In conjunction with the above, the patient exclaimed features consistent with intermittent claudication upon presentation. On examination, the patient was vitally stable; on appearance, the abdomen was not distended, and superficial and deep palpation revealed significant tenderness and guarding in the upper quadrants with a positive rebound sign. Auscultation reported normal bowel sounds and a quick vascular exam revealed strong peripheral pulses with no evidence of acute arterial/venous disease. Upon review, the patient was kept nil per mouth and was started on intravenous (IV) fluids and IV/oral (PO) analgesia. An abdominal and pelvic CT was promptly requested with urgency, which revealed four-quadrant ascites alongside mural thickening and diffuse edema of the jejunum (Figure 1). These findings kept up with peritonitis. In addition, blood examination revealed normal lactate and serum amylase.

The patient had a significant history of pulmonary sarcoidosis confirmed via a biopsy obtained during bronchoscopy and endobronchial ultrasound (EBUS) in 2014. This was managed with prednisolone until remission. In addition, the patient had a history of asthma diagnosed at age seven, which at the time of presentation was well controlled and free of any active therapy. The patient was diagnosed with sarcoid-related arthropathy in 2015, which was being managed via methotrexate and Humira. This regimen was stopped after the development of a right-sided pleural effusion in 2019. The patient had a nil significant past surgical history with the exception of a scaphoid fracture to the right wrist. The patient upon presentation was not on any routine medications, had no known drug allergies, no significant family history, was a non-smoker, and consumed approximately two to three units of alcohol per week.

**Figure 2:**
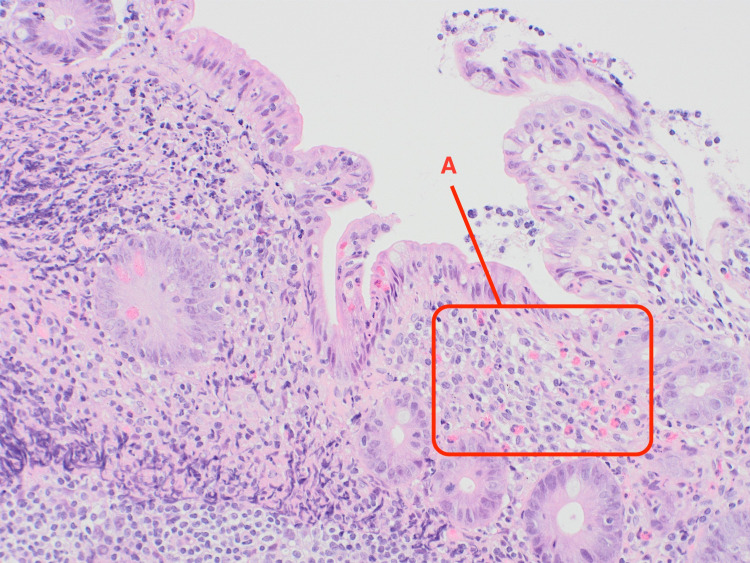
Eosinophils in the mucosa of terminal ileum (examples labeled A).

**Figure 3:**
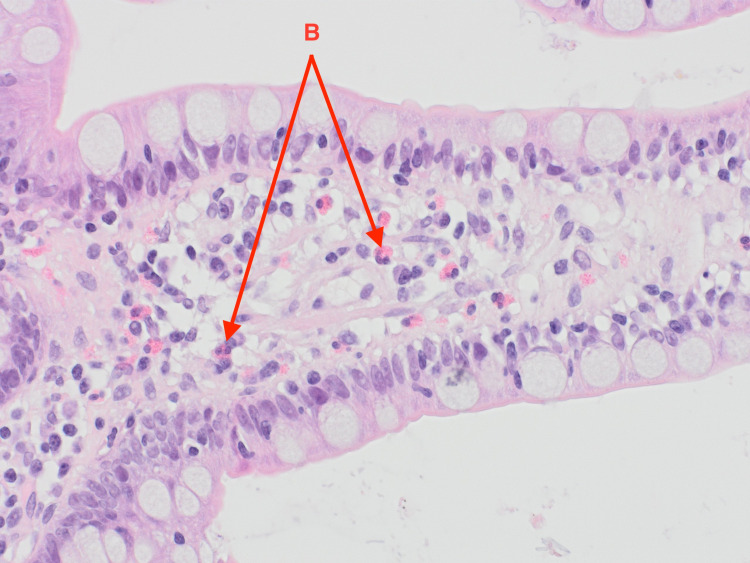
Eosinophils in the lamina propria of terminal ileum villi (examples labeled B).

**Figure 4:**
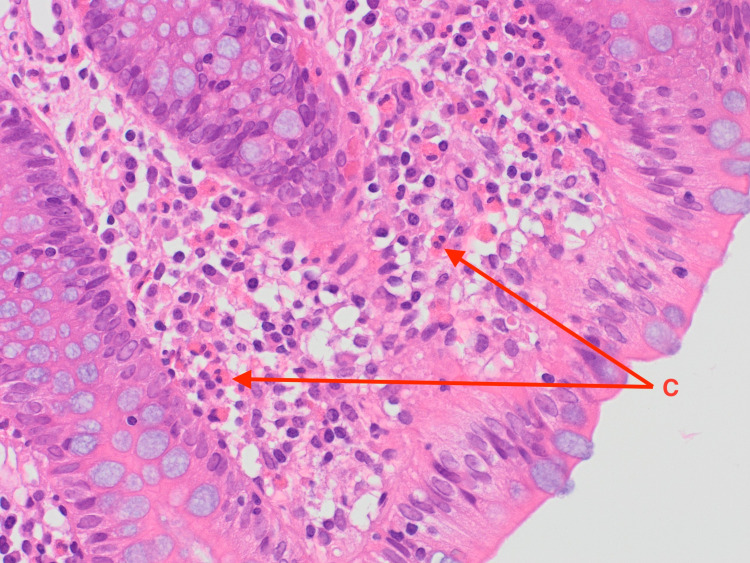
Eosinophils in the right colonic lamina propria (examples labeled C).

**Figure 5:**
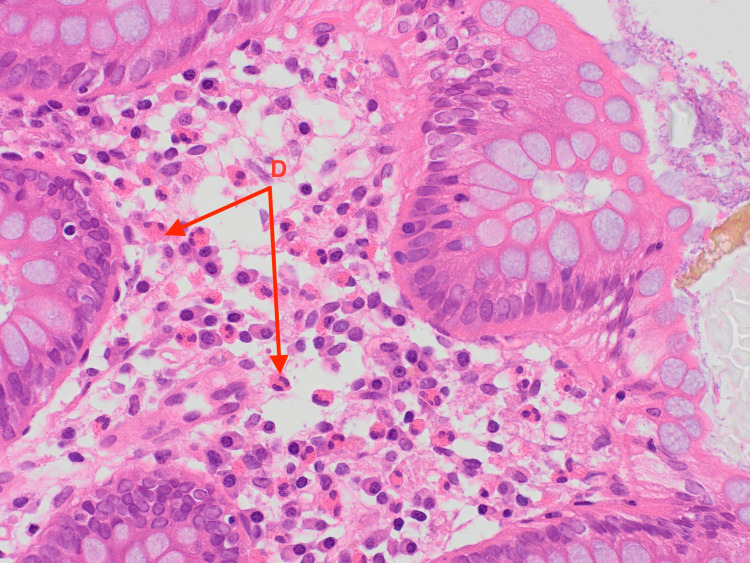
Eosinophils in the right colonic mucosa (examples labeled D).

Following the results of the abdominal and pelvic CT, an exploratory laparotomy was performed and a wedge biopsy of the ileal and right colonic wall was sent for histopathological analysis. Pathology subsequently revealed a florid acute serositis with the marked expansion of the subserosal fatty tissue with a hypereosinophilic cell population identified in the right colonic and terminal ileum mucosa and lamina propria (Figures 2-5). There was no evidence of any microorganism, vasculitis, or malignancy including lymphoma.

Given the presence of a significant eosinophilic cell population in a non-neoplastic setting, pathology recommended investigating two distinct differentials: hypersensitivity eosinophilic type inflammatory process and a parasitic infection given the patient’s travel and occupational history. Immunology and infectious disease were thereby consulted.

In regards to infectious disease, given the patient’s recent travel history to Lebanon, Israel, and Syria, a full parasitic screen was performed to exclude a *Strongyloides* or schistosomal infection. In addition, routine tuberculosis, hepatitis, and HIV screening were mandated, all of which reported nil significant results. An ultrasound of the abdomen was requested as a last resort intervention to visually rule out any evidence of a parasitic infection and/or abscess. Results similarly revealed normal pathology and he was thereby discharged from the infectious disease service. From an immunology standpoint, a serum tryptase was performed and an extensive allergen profiling screen was conducted, which revealed normal outcomes. A hypersensitivity response was therefore unlikely. However, given the patient’s biopsy and CT findings, another pathology worth ruling out was eosinophilic gastritis, as it usually manifests as ascites and peritonitis. A gastroenterology review was warranted, which led to performing a full oesophago-gastro-duodenoscopy (OGD) and colonoscopy to harvest gastric, esophageal, and colonic biopsies to investigate further.

**Figure 6:**
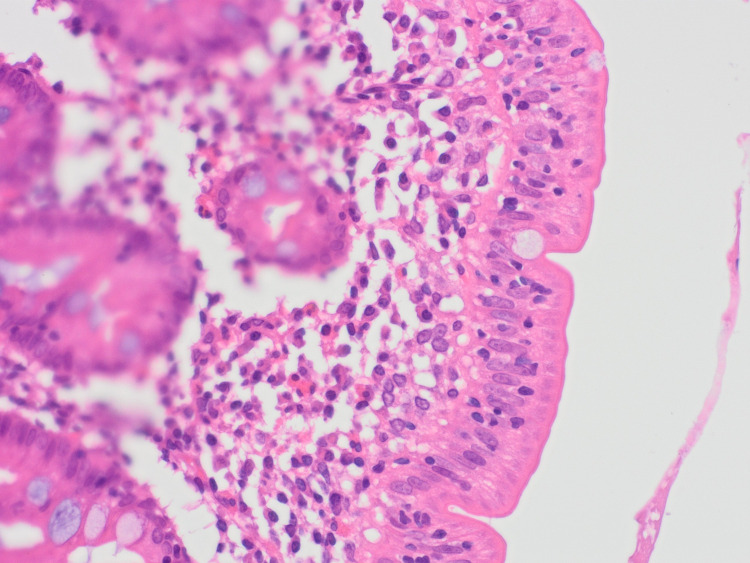
Intraepithelial lymphocytes in D2 duodenum.

**Figure 7:**
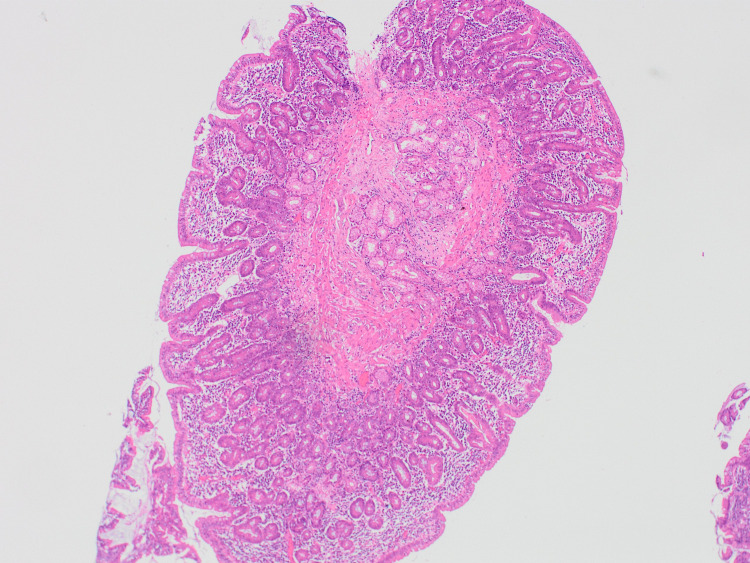
Subtotal villous atrophy in D2 duodenum.

Biopsy revealed the lower esophageal squamous mucosa to have mild features of reflux with normal mucosa in all other sections and no evidence of eosinophilic esophagitis throughout. Gastric biopsy showed mild chronic inflammation in the pylorus, antrum, and fundus with no evidence of significant eosinophilic cell population and no signs of lymphocytic gastritis. Subtotal villous atrophy was identified in the duodenum with increased intraepithelial lymphocytes (Figures 6, 7).No evidence of neoplastic cell infiltrates was identified in any specimens. Moreover, some fragments of the terminal ileum showed reactive type lymphoid cell population, while others showed normal villous outline with no intraepithelial lymphocytes. There appeared to be a mild increase in cellularity in the lamina propria of the left side of the colon. There was no evidence of lymphoproliferative disease, no features to support eosinophilic gastritis, no evidence of mucosal active inflammation, and no evidence of infective or lymphocytic colitis. Regarding the suspicion of infectious pathology, no organism was isolated.

Moreover, upon admission, the patient also complained of symptoms characteristic of intermittent claudication. He reported bilateral exertional calf pain radiating toward the quadriceps, which worsened when jogging on a brief incline. The patient reported having difficulty keeping his legs straight and could only withstand jogging up to 500-600 meters before having to stop due to pain, weakness, and fatigue. This was a drastic change from his previous baseline, which included being able to run up to 5 km with ease. The patient reported no symptoms at rest and denied any restrictions upon walking. Given the patient’s history with sarcoidosis, we were suspicious of sarcoid-related mono/polyneuropathy versus a vascular insult. Given the patient’s age, social history, and active physical status, the more conventional causes of claudication including peripheral arterial disease were extremely unlikely.

A complete neurological assessment was performed, which revealed no signs of numbness or paresthesia. All reflexes were normal, cranial nerves I-XII were intact, the tone was normal, and power was five out of five in all limbs. In addition, Romberg's sign was negative, and cerebellar and sensory exams were normal, as was his gait. There was no clinical sign of motor or sensory neuropathy. Neurology was consulted and an electromyogram (EMG) + nerve conduction study (NCS) was performed for exclusion, which was unremarkable.

Furthermore, claudication was examined from a vascular standpoint. Physical examination revealed low character femoral pulses and an ankle-brachial pressure index (ABPI) was requested, which showed bilaterally reduced values. Subsequently, a diaphragm to lower limb CT angiogram was done alongside a vasculitis panel to exclude a large vessel vasculitis and luminal pathology (dissection, web, endofibrosis, etc.). The patient’s erythrocyte sedimentation rate (ESR) was reported to be very high at 48, anti-myeloperoxidase (anti-MPO) ANCA and anti-PR3 ANCA were negative, C3 and C4 complement screens were normal, and perinuclear ANCA was positive. The CT angiogram similarly revealed an aortoiliac mural thickening keeping with vasculitis more likely to be a secondary reaction to his initial peritonitis. Any luminal pathology was excluded and the patency of all lower limb arteries was guaranteed.

Due to the astronomical ESR levels, CT angiogram findings, and the patient’s history with sarcoidosis, it was thought insights from a rheumatology perspective would be worthwhile. A complete autoimmune screen was performed to include rheumatoid factor (RhF), anti-citrullinated protein (anti-CCP), antinuclear antibody (ANA), and anti-extractable nuclear antigen (anti-ENA) screens, which were all normal.

**Figure 8:**
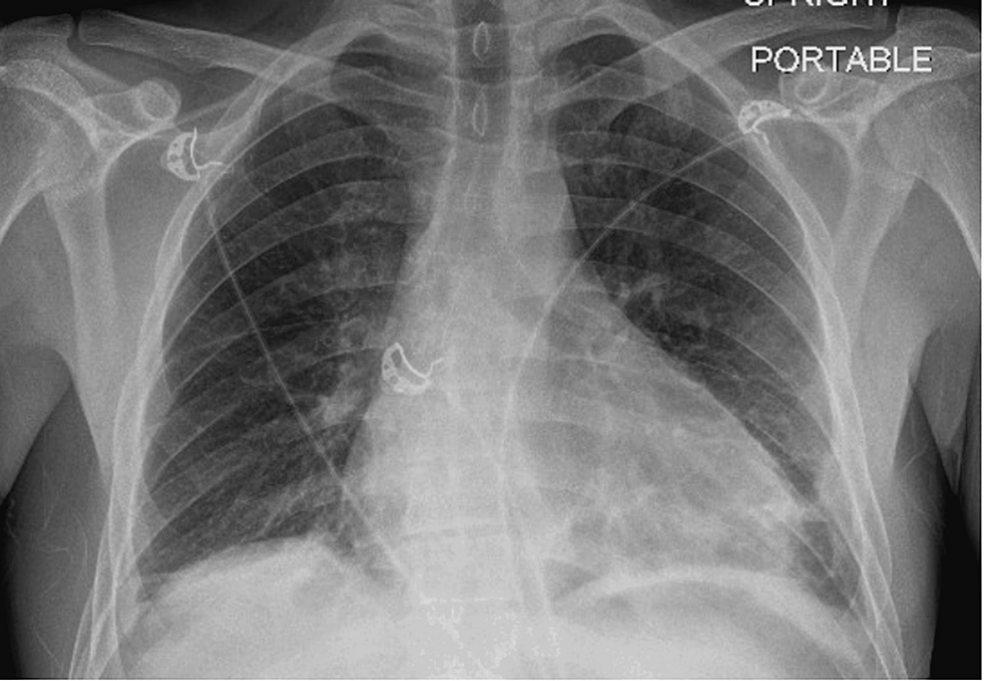
Portable anteroposterior erect film demonstrating new subsegmental atelectasis at right lung base + infiltrates and atelectasis within the left lower lobe and inferior segment of the lingula.

**Figure 9:**
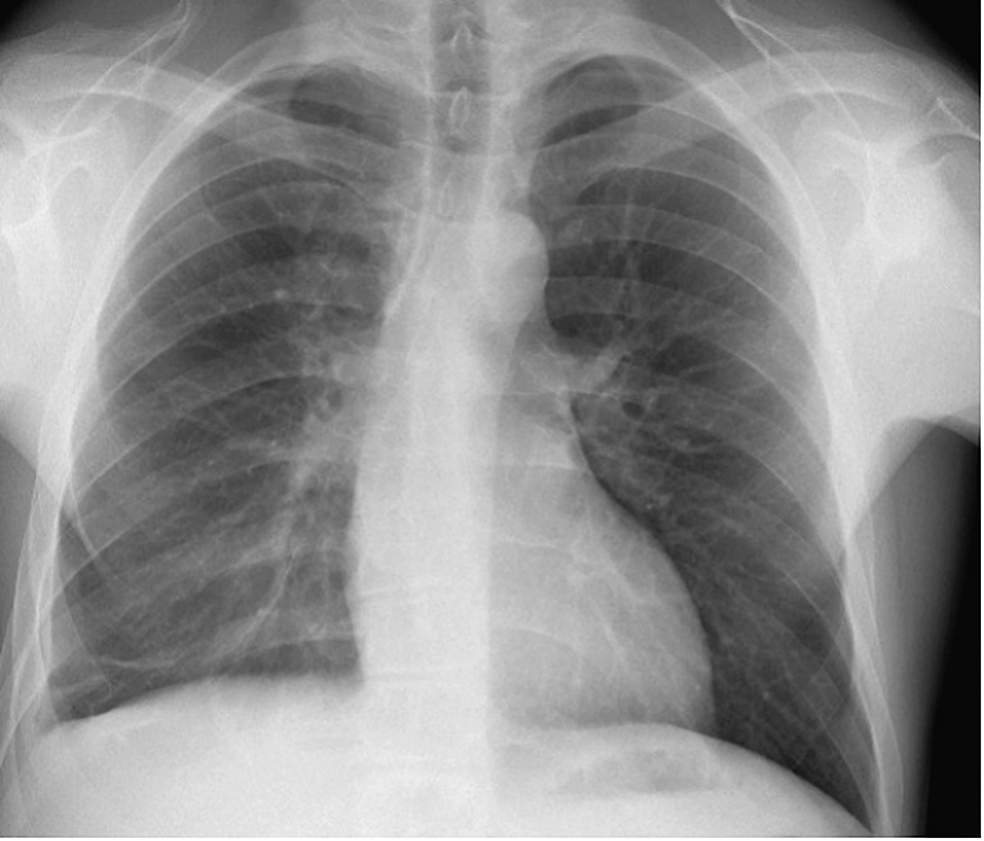
Chest X-ray taken three weeks after the previous film demonstrating resolution of infiltrates previously present on the left lung and new mild infiltrates in the right middle lobe.

Ultimately, a respiratory review was warranted given the patient’s history with pulmonary sarcoidosis and a thorough evaluation sought the need for multiple investigations including a CT sinus, CT thorax, and bronchoalveolar lavage to investigate a preliminary diagnosis of EGPA. In addition, he was commenced on 500 mg of IV methylprednisolone on a once-daily basis for three days followed by a weaning course of prednisolone starting at 60 mg and Septrin as prophylaxis. CT thorax revealed small bilateral pleural effusions, bibasal atelectasis, slightly prominent bilateral hilar, and perihilar lymphadenopathy consistent with sarcoidosis, and a very large 3 cm pericardial effusion, which warranted urgent cardiothoracic input. The patient was thereby admitted to the coronary care unit and started on colchicine. Pericardiocentesis was not performed given the posterior location of the effusion and a cardiac MRI was booked to investigate for any fibrosis/sarcoid following resolution of the effusion. CT sinus revealed a hypoplastic left maxillary sinus with longstanding opacification and bronchoalveolar lavage fluid was shown to have >5% eosinophils. The patient had 22 chest X-rays and three CT thorax scans performed from 2014 to 2022. All chest radiology was reviewed to identify any evidence of migratory infiltrates. Temporal proof showing infiltrates being migratory was identified (Figures 8, 9) with the addition of altering small pleural effusions seen bilaterally (a feature that is evident in 50% of patients with EGPA) [6].

The patient met four out of the six criteria for EGPA as proposed by the 1990 ACR criteria. The only feature he did not exhibit was evidence of mono/polyneuropathy, which was excluded via EMG/NCS and evidence of vascular eosinophilic infiltrates (Table 1). The latter was a significant limitation in the case report because while there was a mild increase in eosinophils in the lamina propria of the ileal and right colon mucosa, these were concluded as non-specific and there were no histological features to suggest granulomatous/eosinophilic vasculitis on the biopsies. However, these biopsies were mucosal only and deeper biopsies that can be diagnostic in this entity were not obtained. In addition, the patient received 10 points as per the revised 2022 ACR/EULAR criteria (Table 2) and was ultimately diagnosed with CCS.

**Table 1:** American College of Rheumatology (ACR) 1990 EGPA diagnostic criteria. EGPA: eosinophilic granulomatosis with polyangiitis; pANCA: perinuclear antineutrophil cytoplasmic antibody; EMG/NCS: electromyogram + nerve conduction study.

ACR 1990 criteria for EGPA	Criteria met	Criteria not met
Asthma	History of asthma at age 7. Asthma is well-controlled and the patient is free of any active therapy	X
Migratory infiltrates in lungs	Migratory infiltrates were noted on multiple chest X-rays	X
Paranasal sinus abnormality	CT sinus: hypoplastic left maxillary sinus + longstanding opacification	X
Mono or polyneuropathy	X	EMG/NCS: normal findings. No indication of neuropathy
Peripheral blood eosinophilia (>10%)	Eosinophils 1.23 x 10^9^/L at the highest point	X
Eosinophilic tissue infiltrates in the biopsy	Full-thickness wedge biopsy of the ileal wall revealed a hypereosinophilic cell population	No evidence of eosinophilic vascular infiltrates was noted. However, deeper biopsies that can be diagnostic were not obtained
Other relevant findings to support EGPA	pANCA positive (identified in 75% of confirmed EGPA cases). Bronchoalveolar lavage fluid revealed >5% eosinophils. Extrapulmonary organ involvement: new pericardial effusion with myocarditis and eosinophilic peritonitis	

**Table 2:** ACR/EULAR 2022 EGPA diagnostic criteria. ACR: American College of Rheumatology; EULAR: European Alliance of Associations for Rheumatology; EGPA: eosinophilic granulomatosis with polyangiitis; ANCA: antineutrophil cytoplasmic antibody; pANCA: perinuclear antineutrophil cytoplasmic antibody; EMG/NCS: electromyogram + nerve conduction study.

ACR/EULAR 2022 criteria for EGPA	Points assigned	Patient features	Points received
Blood eosinophil count ≥ 1 x 10^9/L	+5	The patient had eosinophils of 1.23 x 10^9/L at the highest point	+5
Extravascular eosinophilic-predominant inflammation on biopsy	+2	Full-thickness wedge biopsy of the ileal wall revealed a dominant hypereosinophilic cell population	+2
Positive test for cytoplasmic antineutrophil cytoplasmic antibodies (cANCA) or anti-proteinase 3 (anti-PR3) antibodies	-3	The patient was negative for cANCA and anti-PR3 ANCA. Positive for pANCA	+0
Hematuria	-1	No evidence of hematuria	+0
Obstructive airway disease	+3	History of asthma	+3
Nasal polyps	+3	No evidence of nasal polyps	+0
Mononeuritis multiplex	+1	No evidence. Excluded via EMG/NCS	+0
Total points: 10			

The patient was subsequently started on a course of subcutaneous mepolizumab prior to discharge and reported a dramatic improvement in his symptoms instantly. In regards to long-term management, he was commenced on a monthly dose of 300 mg subcutaneous mepolizumab as maintenance and a tapering course of prednisone starting at 60 mg. Septrin prophylaxis was prescribed until prednisolone was tapered to less than 15 mg once daily and folic acid was given for three months.

As of date, the patient is now four months post commencing therapy for EGPA. He has had four mepolizumab injections, is currently on 10 mg prednisolone, and is thus off the Septrin prophylaxis. The patient reported excellent response to prednisolone and subsequent mepolizumab maintenance and was recently able to run over 5 km for the first time in almost eight months. On physical examination, his chest was clear with some reduced air entry at the right base. His saturations were 97% on room air with a heart rate of 92 beats per minute. His most recent chest X-ray was excellent, demonstrating only minor pleural thickening on the right, which is stable. Recent pulmonary function tests (PFTs) demonstrate a mild restrictive pattern with a forced vital capacity (FVC) of 76% (3.61 L), which has improved from the previous numbers. A cardiac MRI done a month earlier showed the resolution of his pericardial effusion and a significant decrease in eosinophil count. He denies any further abdominal pain, nausea, or vomiting. He also denies dyspnea on exertion and nasal symptoms. He does report reflux, which has improved on twice-daily proton pump inhibitor (PPI), and intermittent discomfort on deep inspiration, which is long-standing. He reports no joint pain. Moving forward, the aim is to reduce prednisolone to 5 mg followed by complete termination of steroids pending assessment for adrenal suppression.

## Discussion

A differential diagnosis worth considering in this circumstance is the hypereosinophilic syndrome (HES). Hypereosinophilia (HE) by definition is defined as an absolute eosinophil count of >1.5 x 10^9/L in the peripheral blood on two separate examinations separated in time by at least one month and/or histological evidence that demonstrates eosinophil dominant inflammation of tissues [7]. In this case, the patient had a maximum eosinophil count of 1.23 x 10^9/L on only one occasion. However, biopsy specimens obtained from the bowel did reveal tissue HE. On the other hand, HES is defined by the association of HE with eosinophil-mediated organ damage, provided other potential causes for the damage have been excluded. This patient had tissue evidence of HE, which presented as eosinophilic peritonitis [7]. Although this patient seems to fit in the HES definition, it is important to investigate further. HES is historically a poorly defined pathology with many attempts made recently to redefine it. There are different types of HES: primary, secondary, and idiopathic. Primary HES is associated with a neoplastic source. Secondary HES is a reactive response, either to a parasite, solid tumor, lymphoma, or other pathology. Lastly, idiopathic HES (IHES) is diagnosed when the underlying cause of HE remains unknown despite thorough etiologic workup. It was determined that HE secondary to a reactive response or neoplasm was excluded through extensive workup [7]. Therefore, it can be debated whether this patient is more suited to be diagnosed with EGPA or IHES. Understandably, diagnosing EGPA, particularly in the absence of documented vasculitis on tissue biopsy is difficult. Some people prefer to view EGPA as an organ-restricted eosinophilic disorder, since it is a disease of one organ (blood vessels), although clinical manifestations can involve the sinuses, lungs, skin, heart, peripheral nerves, and other structures. However, other experts believe it is more appropriately classified as a defined syndrome associated with HE [8]. This issue remains unclear and an area of debate, which is also one of the reasons why awareness regarding this matter should be raised. Moreover, many patients meet diagnostic criteria for both disorders and clinical determination is very hard as patients presenting with EGPA or IHES will present with extremely similar symptoms and signs. However, it is important to note that early stages of EGPA usually reveal eosinophil tissue infiltration without the distinguishing granulomas and frank vasculitis of late-stage EGPA [8]. Therefore, a diagnosis of EGPA cannot be excluded, even if there is no evidence of vasculitis or necrotizing granulomas. These findings are very typical and normal in people with early-stage EGPA as seen in this scenario. Also, both disorders (IHES and EGPA) usually respond initially to glucocorticoids, and while glucocorticoid therapy is beneficial, it may further impede our ability to distinguish between these two conditions. Ultimately, the lack of histological evidence of vasculitis, in this case, is explained by the disease being in its early stages alongside the unavailability of deeper biopsies that can be diagnostic. The patient scored 10 points in concordance with the 2022 ACR/EULAR criteria and 4 with the 1990 ACR criteria. This alongside a myriad of other supporting features points us toward a diagnosis of EGPA more than IHES despite underlying weaknesses.

EGPA can manifest in multiple ways with a wide variety of symptoms. Almost all individuals (97%) with CSS actively have asthma or a history of asthma, which was present in this case. In addition, nearly two-thirds (61%) of patients have some element of atopy including sinusitis and allergic rhinitis, which was absent in this case [9]. Extensive allergen testing was performed as an outpatient as per immunology, which yielded nil significant findings. Moreover, cough and hemoptysis occur in just over a third of individuals (37%), arthralgias occur in 40% of patients, and skin changes occur in half of all patients (49%) [9]. None of these was evident in this case. Cardiac events in CSS include but are not limited to pericardial effusions, myocarditis, heart failure, and myocardial ischemia secondary to coronary vasospasms and/or vasculitis. As represented in this report, a very large pericardial effusion was incidentally found on this patient’s CT thorax, which was successfully treated via a course of colchicine, steroids, and intensive care in coronary care unit. Pericardiocentesis should be considered in most cases but this can be limited by the anatomical position of the effusion. Surprisingly, cardiac involvement is one of the rare but serious manifestations, accounting for approximately 50% of deaths attributable to EGPA [10]. Neuropathy, specifically a mononeuritis complex, can mimic intermittent claudication (IC) and is evident in 77% of cases thus included in the ACR diagnostic criteria for EGPA. In this case, the patient presented with IC, which upon further investigation was concluded to be a reactive vasculitis secondary to the peritonitis. However, a nerve conduction study and electromyography were still conducted for exclusion. Most importantly, CSS manifesting in the gastrointestinal tract is evident in 31% of cases. This usually presents as abdominal pain, as in our case, diarrhea, and occasionally gastrointestinal bleeding [9]. Presentation as an acute abdomen is hypothesized to be correlated to the degree of local inflammation generated by EGPA. In this case, it could be concluded that a severe local inflammation spawned significant peritoneal irritation, producing signs at first local and then diffuse peritonism. In all, there are multiple factors that made this case an atypical presentation of CSS; from the absence of neuropathy to the presence of rare cardiac and GI manifestations.

Treatment is conducted in two phases: initial and maintenance. Initial treatment is recommended to last between six and 12 months before inducing maintenance therapy for a further 12-18 months. Initial therapy involves systemic oral glucocorticoids +/- an additional immunosuppressive agent. The oral glucocorticoid of choice is prednisone dosed between 0.5 and 1 mg/kg per day. However, a three-day regimen of 1 g IV methylprednisolone might be commenced prior to normal oral prednisone in some cases where the acute multiorgan disease is prevalent [11]. Moreover, the need for an additional immunosuppressive agent as part of the initial treatment is decided based on the severity of the disease. This is determined by five criteria making up a five-factor scoring system: (1) age > 65 years; (2) cardiac insufficiency; (3) renal insufficiency; (4) gastrointestinal involvement; and (5) absence of ENT manifestations (presence is associated with a better prognosis) [12]. Individuals scoring above two points warrant the need for an additional immunosuppressive agent in conjunction with prednisone. The agent of choice is cyclophosphamide, which is taken daily if oral or monthly if intravenous. This was not commenced in this case, as the patient did not meet the criteria. Septrin is administered as pneumocystis prophylaxis due to the risk of *Pneumocystis* pneumonia (PCP) in heavily immunosuppressed individuals secondary to high-dose glucocorticoids. Maintenance therapy is commenced once remission is established with glucocorticoids +/- cyclophosphamide. This includes the use of less toxic immunosuppressive medications and a tapering dose of glucocorticoids. A wide variety of agents can be used as maintenance therapies including azathioprine, methotrexate, leflunomide, and anti-interleukin-5 antibodies like mepolizumab. Given its very modest side effect profile, the lower reported rate of adverse events, and proven efficacy based on multiple multicenter trials, a monthly course of 300 mg subcutaneous mepolizumab is the recommended agent to be used in conjunction with a weaning course of prednisone as best maintenance therapy for EGPA [13].

Management of EGPA, in this case, was evidence-based using up-to-date guidelines. The patient was commenced on 1 g IV methylprednisolone once daily for three days followed by 60 mg prednisone and prophylactic Septrin as initial therapy. This was administered until remission. A monthly 300 mg subcutaneous mepolizumab was then commenced alongside a tapering course of steroids. Four months down the line, the patient is now on 10 mg prednisone, off the Septrin prophylaxis, and due to start a tapered dose of 5 mg prednisone in due time followed by complete steroid elimination.

EGPA has a relatively good prognosis in recent times with a 70-90% survival rate after five years [14]. The mortality is positively correlated to the severity of the disease, which as previously stated is calculated by the Five-Factor Score (F5S). Of the five factors, cardiac involvement, GI disease, and an age greater than 65 years appear to be the strongest indicators of poor prognosis [15]. Most mortalities usually result from the vasculitic stage of the disease with cardiac involvement (heart failure/myocardial infarction) being the leading cause of EGPA-associated deaths followed by cerebral hemorrhage, renal failure, GI bleeding, and status asthmaticus. In addition, those with a positive anti-MPO ANCA are at a higher risk of relapse compared to anti-MPO negativity [15].

## Conclusions

Ultimately, we present a diagnostically challenging and atypical case of EGPA presenting as an acute abdomen, which required very extensive investigation, multidisciplinary consultation, and laparotomy to achieve a diagnosis. Along the way, we have learned the importance of multidisciplinary team involvement in investigating rare presentations, the fruits of a thorough evaluation, and the need for open-mindedness in surgery to broaden differentials to include medical pathologies.
